# Metabolic Reprogramming and Potential Therapeutic Targets in Lymphoma

**DOI:** 10.3390/ijms24065493

**Published:** 2023-03-13

**Authors:** Yuyang Pang, Tingxun Lu, Zijun Y. Xu-Monette, Ken H. Young

**Affiliations:** 1 Division of Hematopathology, Department of Pathology, Duke University School of Medicine, Durham, NC 27710, USA; 2 Department of Hematology, Ninth People’s Hospital, Shanghai Jiao-Tong University School of Medicine, Shanghai 200025, China; 3 Duke Cancer Institute, Durham, NC 27710, USA

**Keywords:** lymphoma, metabolism, targeted therapy

## Abstract

Lymphoma is a heterogeneous group of diseases that often require their metabolism program to fulfill the demand of cell proliferation. Features of metabolism in lymphoma cells include high glucose uptake, deregulated expression of enzymes related to glycolysis, dual capacity for glycolytic and oxidative metabolism, elevated glutamine metabolism, and fatty acid synthesis. These aberrant metabolic changes lead to tumorigenesis, disease progression, and resistance to lymphoma chemotherapy. This metabolic reprogramming, including glucose, nucleic acid, fatty acid, and amino acid metabolism, is a dynamic process caused not only by genetic and epigenetic changes, but also by changes in the microenvironment affected by viral infections. Notably, some critical metabolic enzymes and metabolites may play vital roles in lymphomagenesis and progression. Recent studies have uncovered that metabolic pathways might have clinical impacts on the diagnosis, characterization, and treatment of lymphoma subtypes. However, determining the clinical relevance of biomarkers and therapeutic targets related to lymphoma metabolism is still challenging. In this review, we systematically summarize current studies on metabolism reprogramming in lymphoma, and we mainly focus on disorders of glucose, amino acids, and lipid metabolisms, as well as dysregulation of molecules in metabolic pathways, oncometabolites, and potential metabolic biomarkers. We then discuss strategies directly or indirectly for those potential therapeutic targets. Finally, we prospect the future directions of lymphoma treatment on metabolic reprogramming.

## 1. Introduction

The reprogramming of metabolic pathways is shaped by both oncogenes and tumor-suppressor genes to ensure a stabilized supply of intermediate metabolites for the biosynthesis of biomass and generation of ATP in tumor cells [1,2,3,4,5,6]. Deregulated uptake of glucose and amino acids (AA), using nutrients and intermediates of the tricarboxylic acid cycle (TCA cycle) occasionally, NADPH production, increased requirements for nitrogen, alterations in metabolite-driven gene regulation, and interactions between the metabolism and microenvironment are regarded as crucial hallmarks of tumor metabolism [7,8].

Lymphoma is a group of heterogeneous diseases, and 40–50% of patients achieve a complete response after inductive therapy [9,10,11]. High glucose uptake, deregulated expression of glycolytic enzymes, the dual ability for both glycolytic and oxidative metabolism, active glycolysis prevailing over aerobic glucose metabolism without defects in mitochondrial function, and increased glutamine metabolism and fatty acid biosynthesis are notable metabolic effects of lymphoma [12]. This metabolic reprogramming is a dynamic process involving dysregulation of transcription factors and signaling pathways, such as phosphatidylinositol 3-kinase (PI3K)/mammalian target of rapamycin (mTOR), hypoxia-inducible factors (HIF), MYC, p53, and AMP-activated protein kinase (AMPK). Additionally, some viruses, such as Epstein–Barr virus (EBV), also contribute to the metabolic abnormalities associated with lymphoma. Changes in the tumor microenvironment—hypoxia, for example—can also induce metabolic alterations in lymphoma. Under hypoxic conditions, several pathways of cell survival and proliferation, such as glycolysis, glutamine oxidation, bioenergetics, and redox homeostasis, are activated. These metabolic alterations are associated with both tumorigenesis and disease progression [13]. Some dominant mutations in genes encoding metabolic enzymes, which play significant roles in tumorigenesis and tumor progression, cannot be ignored. Thus, therapeutic strategies targeting critical metabolic enzymes, transcription factors, signaling pathways, and the microenvironment are being investigated both in preclinical and clinical research.

In this review, we focus on the metabolic characteristics of lymphoma. We aim to highlight the dysregulation and functional defects in metabolic pathways in lymphoid malignancies and discuss metabolic biomarkers and targeted therapies.

## 2. Metabolic Alterations in Lymphoma

### 2.1. Altered Glucose Metabolism

#### 2.1.1. Up-Regulated Glucose Uptake

**Figure 1:**
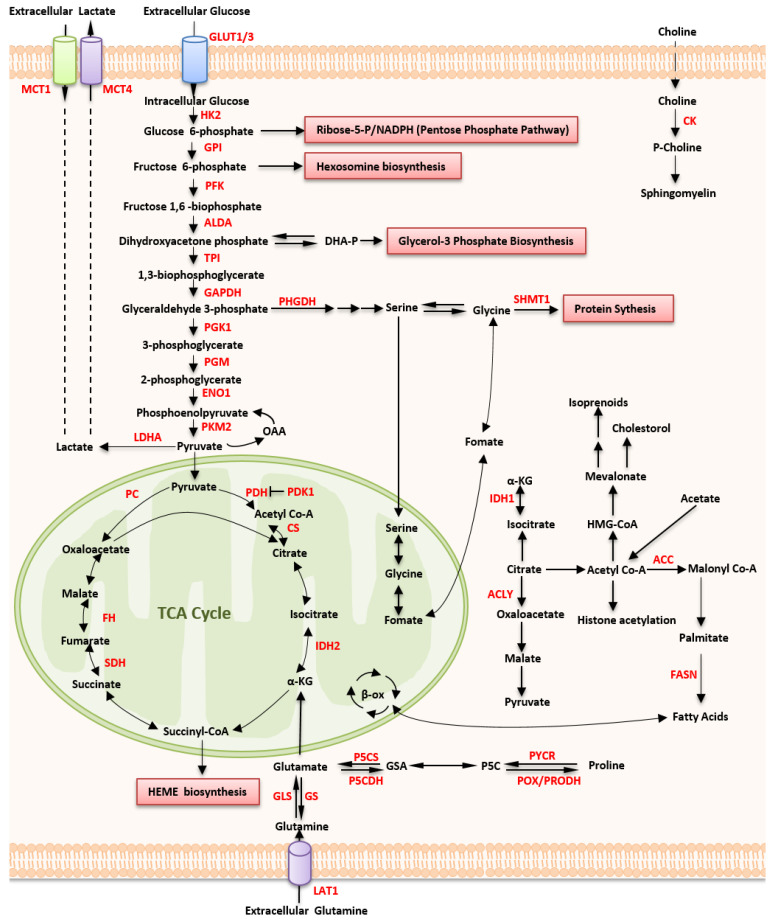
Metabolic pathways in lymphoma. Lymphoma cells take up glucose and glutamine also to fuel the tricarboxylic acid (TCA) cycle, oxidative phosphorylation (OXPHOS), the pentose phosphate pathway (nucleotide synthesis), and the syntheses of hexosamine (involved in the synthesis of glycosylated molecules), amino acids (proteins), and lipids. Lymphoma cells are also able to take up lactate, free fatty acids, and ketones released predominantly by surrounding catabolic cells, which can be used to replenish TCA-cycle intermediates and fuel OXPHOS. Together, these pathways generate sufficient levels of cellular components to support cell proliferation (necessary nutrient transporters and metabolic enzymes are highlighted in red). Abbreviations: MCT1, monocarboxylate transporter 1; MCT4, monocarboxylate transporter 4; GLUT1/GLUT4, glucose transporter 1/4; HK2, hexokinase; GPI, glucose-6-phosphate isomerase; PFK, phosphofructokinase; ALDA, aldolase A; TPI, triosephosphate isomerase; GAPDH, glyceraldehyde-3-phosphate dehydrogenase; PHGDH, phosphoglycerate dehydrogenase; SHMT1, serine hydroxymethyltransferase 1; PGK1, phosphoglycerate kinase 1; PGM, phosphoglycerate mutase; ENO1, enolase-1; PKM2, pyruvate kinase M2; LDHA, lactate dehydrogenase A; PDH, pyruvate dehydrogenase; PDK1, pyruvate dehydrogenase kinase 1; PC, pyruvate carboxylase; CS, citrate lyase; IDH1/2, isocitrate dehydrogenase1/2; GLS, glutaminase; GS, glutamine synthetase; SDH, succinate dehydrogenase; FH, fumarate hydratase; P5CS, Δ (1)-pyrroline-5-carboxylate (P5C) synthase; P5CDH, P5C dehydrogenase; LAT1, L-type amino-acid transporter 1; CK, choline kinase; ACLY, ATP citrate lyase; ACC, acetyl CoA carboxylase; FASN, fatty acid synthase; PYCR, pyrroline-5-carboxylate reductase; POX/PRODH, proline dehydrogenase; TCA, tricarboxylic acid.

High glucose uptake is one of the hallmarks of aggressive lymphomas and is associated with poor outcomes in diffuse large B-cell lymphoma (DLBCL) [14,15]. Increased glucose uptake is a feature of malignant cells and can be detected via positron emission tomography/computed tomography (FDG-PET), a non-invasive molecular imaging tool, by analyzing glucose metabolism [16]. FDG-PET has become a standard staging and efficacy evaluation method, especially for DLBCL [7]. Hexokinase 2 (HK2) and, glucose transporter (GLUT) 3 contribute to FDG uptake and higher HK2 expression in DLBCL may contribute to its higher FDG uptake than non-DLBCL [17] (Table 1). GLUT3 was also identified as a glucose transporter of FDG accumulation in primary central nervous system lymphoma [18] (Table 1). In patients with advanced-stage classic Hodgkin lymphoma (cHL), however, higher GLUT1 expression is associated with programmed cell death ligand 1 (PD-L1) and PD-L2 expression and prolonged survival [19] (Table 1, Figure 1).

#### 2.1.2. Aerobic Glycolysis

The increased metabolism of glucose to lactate under aerobic conditions (the Warburg effect) is another feature of cancer cells [6]. Compared to oxidative phosphorylation (OxPhos), which produces 36 ATP molecules per glucose molecule, glycolysis is inefficient and generates only two ATP molecules per glucose molecule. However, aerobic glycolytic metabolism represents ~60% of the energy produced in malignant cells due to their uptake of larger amounts of glucose and higher rates of glycolysis [20]. Some crucial glycolytic enzymes are increased and play essential roles in energy metabolism, which are recognized as potential biomarkers and relevant therapeutic targets in cancers, including aggressive B-cell lymphomas [21,22,23]. HK2 is highly expressed in DLBCL and results in higher FDG uptake [17]. HK2 is also required to promote the growth of DLBCL cells under hypoxia [24] (Table 1). Increased levels of serum lactate dehydrogenase (LDH) in primary cutaneous lymphoma (PCL) have been associated with independent risk factors for progression-free survival (PFS) rate and overall survival (OS) rate [25] (Table 1). The LDH-5 is upregulated in non-Hodgkin lymphoma (NHL), which is directly related to clinical stage, extra-nodal site involvement, and performance status of patients with NHL [26] (Table 1). The excreted lactate is eliminated from the cells via monocarboxylate transporters (MCTs), which have been found to be highly expressed in stromal cells of DLBCL samples [27]. Accordingly, therapeutic strategies that disrupt lactate transport might be promising approaches for treating lymphoma [28,29,30,31] (Figure 1).

**Table 1:** Enzymes and metabolite alterations in lymphoma.

Enzymes /Metabolites	Subtype of Lymphoma	Alteration	Mechanism	Ref
GLUT3	DLBCL	upregulated	FDG uptake	[17]
GLUT3	PCNSL	upregulated	glucose transporter of FDG accumulation	[18]
GLUT1	cHL	upregulated	better clinical outcomes	[19]
HK2	DLBCL	upregulated	FDG uptake, promote cell growth	[17,24]
LDH	PCL	upregulated	aggressive clinical behavior, resistance to chemotherapy, and low survival rates	[25]
LDH-5	NHL	highly upregulated	correlated with HIF-1α cytoplasmic accumulation in NHL cells	[26]
MCT1	DLBCL	highly expressed	an OXPHOS phenotype	[27]
SHMT	DLBCL		make glycine for purine synthesis	[32]
IDO 1	DLBCL		converts tryptophan into kynurenine-pathway metabolites, inhibits T-cell activity, and induces immune tolerance, predict a favorable prognosis	[33]
IDO 1	HL		converts tryptophan into kynurenine-pathway metabolites, inhibits T-cell activity, and induces immune tolerance, predict dismal clinical outcomes	[34]
FASN	DLBCL	overexpressed	aggressive clinical course and therapeutic resistance, shorter OS and PFS	[35]
PI3K/AKT/mTOR	BCL and TCL	activated	related to p53, HIF-1α, and MYC	[36,37,38,39]
PKM2	ALCL	inactivated	induces oncogenesis by phosphorylating nuclear STAT3 and regulating the transcription of genes involved in cell survival and proliferation	[40]
POX/PRODH	MYC-driven lymphoma	expressed	mitochondrial tumor suppressor	[41]
GLS	BL	upregulated	enhances glutamine uptake and glutamine catabolism	[41]
Chokα	TCL	overexpressed	regulation of choline metabolism	[42]
SDH	BL	reduced expression	increased the intracellular drug concentration	[43]
IDH2	AITL	mutated	increasing DNA hypermethylation of gene promoters	[44,45]
D2HGDH	DLBCL	mutated	affects histone and DNA methylation and HIF-1α hydroxylation	[46]

Abbreviations: GLUT1, glucose transporter 1; GLUT3, glucose transporter 3; HK2, hexokinase 2; LDH, lactate dehydrogenase; MCT, monocarboxylate transporter; SHMT, serine hydroxymethyltransferase; IDO, indoleamine 2,3-dioxygenase; FASN, fatty acid synthase; PI3K/AKT/mTOR, phosphatidylinositol 3-kinase/Akt serine/threonine kinase 1/mammalian target of rapamycin, PKM2, pyruvate kinase M2; POX/PRODH, proline dehydrogenase; GLS, glutaminase; Chokα, choline kinase-α; SDH, dehydrogenase; IDH2, isocitrate dehydrogenase 2; D2HGDH, D2-hydroxyglutarate dehydrogenase; DLBCL, diffuse large B-cell lymphoma; PCNSL, primary central nervous system lymphoma; cHL, classic Hodgkin lymphoma; PCL, primary cutaneous lymphoma; NHL, non-Hodgkin lymphoma; HL, Hodgkin lymphoma; BCL, B-cell lymphoma; TCL, T-cell lymphoma; ALCL, anaplastic large-cell lymphoma; BL, Burkitt lymphoma; AITL, angioimmunoblastic T-cell lymphoma; FDG, fluorodeoxyglucose; OS, overall survival; PFS, progression free survival; OXPHOS, oxidative phosphorylation; HIF-1α, hypoxia-inducible factors-1 alpha; STAT3, signal transducer and activator of transcription 3.

#### 2.1.3. Mitochondrial Metabolism

As the main metabolic reaction, mitochondrial metabolism reprograms to reserves functions through several mechanisms during aerobic glycolysis. Because it supports bioenergetics and homeostasis, mitochondrial-dependent metabolic reprogramming is crucial for the survival and proliferation of tumor cells during Warburg metabolism [47,48,49] (Figure 1). Although impaired mitochondrial respiration is often construed as the cause of aerobic glycolysis, increasing evidence suggests that both glycolytic and oxidative metabolism exist in tumor cells [50,51], varying among tumor types [20]. Moreover, the fact that glycolysis overwhelms, rather than reduces, mitochondrial function depends on the glycolytic phenotype of the tumor cells [52].

Mitochondria generate most of ATP in lymphoma cells [14]. Glycolytic ATP production differs in DLBCL subtypes with distinct gene expression profiles [15]. Using genome-wide arrays, DLBCL can be subdivided into subsets, mainly including the B cell receptor/proliferation cluster (BCR-DLBCL) and the oxidative phosphorylation cluster (OXPHOS-DLBCL) [53,54,55]. Approximately 30% ATP is produced from glycolysis in cultured OXPHOS-DLBCL cell lines and 45% in BCR-DLBCL cell lines [15]. BCR-DLBCLs have exhibited higher glycolytic flux due to the increased expression of BCR signaling components triggered in a PI3K-dependent manner, making them sensitive to BCR signaling inhibitors.

In contrast, nutrient and energy metabolism in OXPHOS-DLBCL show a significant mitochondrial characteristic with increased activity of components of the mitochondrial electron transport chain and elevated expression of mitochondrial translation factors. Accordingly, OXPHOS is elevated, and there is an increased contribution of the mitochondria to the total cellular energy budget; several glucose-derived carbons and fatty acids are incorporated into the TCA cycle [56,57]. Thus, increased lipogenesis from these carbon substrates is a specific metabolic characteristic of OXPHOS-DLBCL. Although the utilization of fatty-acid-dependent carbons in OXPHOS-DLBCL occurs in parallel to BCR signaling, the former does not display the complete phosphorylation pattern and is resistant to inhibitors of BCR signaling [53,57,58,59]. In contrast, pharmacological inhibition of the mitochondrial translation signal leads to the inhibition of cell proliferation in OXPHOS-DLBCL [56].

### 2.2. Altered Amino Acid Metabolism

Enhancement of glucose metabolism has been assumed to promote the bioenergetic requirements of tumor cells. However, hyperproliferative tumor cells need additional biosynthetic processes beyond glucose metabolism. Therefore, anaplerosis via pyruvate carboxylation, glutamine metabolism, and other sources, such as fatty acid metabolism, is necessary [60,61,62,63,64]. Under aerobic conditions, glutaminolysis, which is regulated by the activity of the MYC oncogene, may play a considerable part in the biosynthetic pathways [63,64].

#### 2.2.1. Glutamine Metabolism

Glutamine is a non-essential and abundant AA in the human body. It is a major nitrogen donor in the biosynthesis of AA and nucleotides [65] and is essential for the survival and proliferation of malignant cells [2]. Glutamine catabolism (called glutamine decomposition) is upgraded in several glutamine-dependent tumors and is often driven by glutaminase (GLS), which transforms glutamine into glutamate and ammonia [4]. In glutamine-dependent tumors, glutamine carbon is present in aspartate, glutamate, and TCA cycle metabolites through glutaminolysis [60,66,67]. DLBCL consumes mitochondrial pyruvate via glutamate-pyruvate transaminase 2 to enable α-ketoglutarate (α-KG) production as part of glutaminolysis [68]. Thus, glutamine exceeds pyruvate as a carbon source for the TCA cycle in DLBCL. Under states of glutamine starvation, glutamine promotes tumor cell proliferation by providing α-KG (a process termed anaplerosis) to supplement depleted TCA cycle intermediates [67,69,70]. In ibrutinib-resistant MCL cells, GLS is overexpressed, and it correlates well with elevated glutamine dependency and glutaminolysis. In addition, GLS expression is correlated with MYC expression and depletion of GLS, or else glutamine significantly reduced cell growth [71]. The nuclear factor erythroid 2-related factor 2 (NFE2L2, also known as NRF2) is a transcription factor that stimulates the expression of genes, and it is a cellular protector that applies to both normal cells and malignant cells [72]. In lung adenocarcinoma, mutations in Kelch-like ECH-associated protein 1 (KEAP1), which constitutively activates NRF2, are required for glutathione synthesis [3]. Keap1 and Nrf2 expression was associated with high international prognostic index (IPI) scores and advanced clinical stage in DLBCL [73]. It was reported that NRF2 plays a significant role in chemotherapy resistance in hematologic malignancies. NRF2 activation metabolically rewires and elevates pathways involved in glutamine metabolism, making it vulnerable to glutaminase inhibition [3,74,75]. In a MYC-induced human Burkitt lymphoma (BL) cell line, glutamine was imported and metabolized through the TCA cycle under hypoxia. Inhibiting glutamine entry into the TCA cycle can inhibit tumor progression [47,76]. Although glutamine supports tumorigenesis directly, glutamine uptake is often severely restricted in developing tumors [77] (Figure 1).

#### 2.2.2. Proline Metabolism

Proline is the only proteinogenic secondary AA that participates in redox regulation and metabolic signaling. Glutamate generated by proline can be further converted to α-KG or can generate glutamine via glutamine synthetase (GS). Proline dehydrogenase (POX/PRODH) participates in the first step of proline catabolism and is considered a mitochondrial tumor suppressor in MYC-driven lymphoma [41] (Table 1). Proline catabolism induced by POX/PRODH generates electrons to produce reactive oxygen species (ROS) and triggers a broad spectrum of downstream reactions, including cell cycle arrest and initiation of apoptosis [41]. In a human BL cell line, MYC suppressed POX/PRODH expression primarily through up-regulating miR-23b*. Furthermore, MYC markedly increased the enzymes of proline biosynthesis from glutamine [41]. Understanding the relationship between glutamine and proline metabolism might enable us to develop novel therapeutic strategies.

#### 2.2.3. Serine, Glycine, and Serine Hydroxymethyltransferase

Serine and glycine are non-essential AAs involved in various anabolic processes to support tumor cell proliferation [78]. Restricting of dietary serine and glycine can decrease tumor growth in *MYC*-driven lymphoma animal models [79]. Serine hydroxymethyltransferase (SHMT) transforms serine into glycine and transfers a one-carbon unit to s tetrahydrofolate. DLBCL cells are defective in glycine uptake, therefore depending on SHMT to make glycine for purine synthesis, which renders them particularly sensitive to SHMT inhibitors [32] (Table 1, Figure 1).

#### 2.2.4. Indoleamine 2,3-Dioxygenase 1

Indoleamine 2,3-dioxygenase 1 (IDO 1) is an intracellular enzyme that converts tryptophan into kynurenine-pathway metabolites, inhibits T-cell activity, and induces immune tolerance in the tumor microenvironment. Increased IDO-positive cells can predict a favorable prognosis in patients with DLBCL treated with R-CHOP [33] (Table 1). However, IDO expression is associated with dismal clinical outcomes in HLs [34] (Table 1). In adult T-cell lymphoma (TCL) and cutaneous T-cell lymphoma (CTCL), IDO may also cause immunosuppression and predict prognosis, which indicates the potential of developing novel cancer immunotherapies targeting IDO [80,81]. Epacadostat, an IDO1 inhibitor, can significantly decrease the concentration of kynurenine in cell cultures [80]. Because of the inhibition of IDO on chimeric antigen receptor T (CD19-CART) cells, fludarabine and cyclophosphamide are used frequently before CART cell administration of CART cells, which can downregulate IDO expression and improve the antitumor activity of CD19-CART cells in vivo [82].

### 2.3. Altered Lipid Metabolism

Elevated fatty acid biosynthesis is one of the hallmarks and an important metabolic inducer in multiple malignancies.

#### 2.3.1. Fatty Acid Synthase

Fatty acid synthase (FASN) plays a critical role in the pathogenesis of lymphoma. Increased expression of FASN is seen in patients with DLBCL and is associated with shorter OS and PFS. Moreover, FASN overexpression in DLBCL is recognized as an independent prognostic factor that predicts an aggressive clinical course and therapeutic resistance [83,84] (Table 1). FASN can induce S6Kinase to facilitate the formation of the USP11-eukaryotic initiation factor 4B (eIF4B) complex for sustained tumorigenic translation in DLBCL. In contrast, eIF4B expression can be reduced at transcription and translation levels when FASN expression is suppressed [83]. FASN is up-regulated in a PI3K-dependent manner in primary effusion lymphoma (PEL) and other types of non-viral B-NHLs [85] (Table 1). FASN is highly and consistently expressed in mantle cell lymphomas (MCLs), as well [86]. Thus, FASN might be a potential therapeutic target for lymphoma therapy. Although inhibiting FASN alone or in combination with PI3K demonstrated a robust decrease in tumor growth, current FASN inhibitors have limited clinical applications because of certain pharmacological limitations [35,87,88] (Table 1, Figure 1).

#### 2.3.2. PPARδ

Peroxisome proliferator-activated receptor delta (PPARδ) is a member of the lipid-activated nuclear receptor family, which regulates the expression of genes involved in glucose, lipid, and cholesterol metabolism [89,90,91,92]. PPARδ can improve metabolic efficiency and tolerance to oxidative stress to make malignant B cells survive under cytotoxic drugs or low glucose and oxygen [91]. PPARδ also regulates the signal transduction process by altering cholesterol metabolism and cytokine signaling. Thus, PPARδ antagonists may also play a therapeutic role in lymphoma [93].

#### 2.3.3. Cholesterol Synthesis

Cancer cells have an increased demand for cholesterol. By targeting and blocking cholesterol uptake, cellular cholesterol can be reduced through the high-affinity, high-density lipoprotein (HDL) receptor scavenger receptor type B-1 (SCARB1). In activated B cell (ABC) DLBCL cell lines, enhanced BCR signaling and the resultant de novo cholesterol synthesis can significantly reduce the ability of HDL-like nanoparticles (HDL NPs) to reduce cellular cholesterol levels, and induce cell death. Thus, ABC DLBCLs are thought to be less sensitive to HDL NPs than GCB DLBCLs. Therefore, by targeting both cellular cholesterol uptake and BCR-associated cholesterol synthesis, cellular cholesterol reduction and apoptosis can be achieved in resistant ABC DLBCL cell lines [94].

#### 2.3.4. Choline Metabolism

Choline kinase-α (Chokα), an enzyme involved in the regulation of choline metabolism, leads to the phosphorylation of choline to phosphocholine [95]. The overexpression of Chokα is frequently observed in cancers and is associated with insufficient histological differentiation and poor prognosis [96]. Dysregulation of choline metabolism in TCLs is related to the overexpression of Chokα in tumor cells [42]. Chokα, as a regulatory gene, possesses carcinogenic activity and could be a potential therapeutic target for hematological malignancies [42] (Table 1 and Table 2).

## 3. Regulation of Altered Metabolism in Lymphoma

Metabolic reprogramming in lymphoma, including glucose metabolism, fatty acid synthesis, and glutaminolysis, is a dynamic process driven by the dysregulation of HIF, MYC, p53, PI3K/mTOR, and AMPK signaling pathways, as well as EBV.

### 3.1. HIF

HIF-1α is a subunit of a heterodimeric transcription factor HIF-1that controls the expression of more than 200 genes affecting large-scale cellular processes, including glucose transport, cellular metabolism, angiogenesis, cell proliferation, apoptosis, and erythropoiesis, in response to cellular hypoxia [97,98,99,100]. In addition, PI3K/AKT/mTOR, RAS-mitogen-activated protein kinase, extracellular signal-regulated kinase, erbB2 families, MYC, and ROS can also independently increase the expression of HIF-1α expression [101,102,103,104,105]. In lymph node biopsies of DLBCL and FL patients, stabilization and upregulation of HIF-1α were observed [106]. Furthermore, in patients with DLBCL treated with R-CHOP-like regimens, the expression of HIF-1α protein is a significant independent prognostic factor for superior survival [107,108].

HIF-1α can also regulate the expression of genes encoding essential enzymes of aerobic glycolysis in BLs [65,109,110,111]. In a MYC-driven BL cell line, HIF-1α, together with MYC, regulates the HK2 and PDK1 expression [112]. Lymphoblastoid cells also showed a Warburg effect regulated by HIF-1α [109,112]. In anaplastic lymphoma kinase (ALK)-rearranged anaplastic large-cell lymphoma (ALCL), hypoxia pathways, regulated by ALK via STAT3- and C/EBPβ-dependent transcription, are significantly enriched [113]. HIF-1α is dispensable for cell growth in ALCL, whereas HIF-2α is required for both cell growth and internal environment maintenance in ALCL [114]. Moreover, HIF-1α plays a crucial role in the pathogenesis of PEL, an aggressive B-cell lymphoma with a poor prognosis caused by KSHV, which promotes the activity of HIF-1α and, in turn, activates the KSHV gene. HIF-1α can also induce aerobic and anaerobic glycolysis and lipid biosynthesis in PEL cell lines [115].

**Figure 2:**
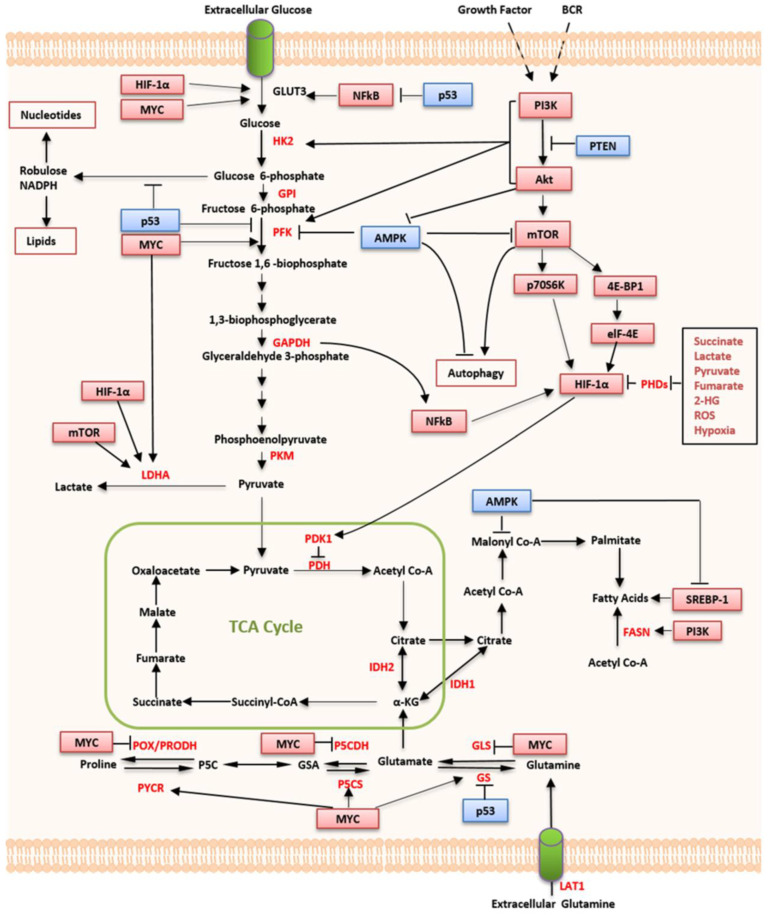
Regulation of lymphoma metabolism by oncogenes and tumor suppressors. Oncogenic signaling (labels in red) regulates the acquisition of abundant nutrients, including glucose and glutamine, and their utilization to support biosynthetic pathways. Intermediates from glycolysis and the TCA cycle supply biosynthetic pathways to produce the macromolecules necessary for cell proliferation. Tumor suppressors (in blue), such as AMPK, p53, and PTEN, act at various nodes to oppose biosynthetic metabolism. Abbreviations: HIF-1α, hypoxia-inducible factor 1 alpha; mTOR, mammalian target of rapamycin; LDHA, lactate dehydrogenase A; GLUT3, glucose transporter 3; HK2, hexokinase; GPI, glucose-6-phosphate isomerase; PFK, phosphofructokinase; GAPDH, glyceraldehyde-3-phosphate dehydrogenase; PKM, pyruvate kinase M; PDK1, pyruvate dehydrogenase kinase 1; PDH, pyruvate dehydrogenase; TCA, tricarboxylic acid; IDH1/2, isocitrate dehydrogenase1/2; POX/PRODH, proline dehydrogenase; PYCR, pyrroline-5-carboxylate reductase; P5C, Δ (1)-pyrroline-5-carboxylate; P5CS, P5C synthase; P5CDH, P5C dehydrogenase; GSA, glutamic semialdehyde; GS, glutamine synthetase; GLS, glutaminase; LAT1, L-type amino-acid transporter 1; FASN, fatty acid synthase; PI3K, phosphatidylinositol 3-kinase; SREBP-1, sterol-regulatory element binding protein-1; AMPK, 5ʹ-AMP-activated protein kinase; NF-κB, nuclear factor kappa B; ROS, reactive oxygen species; 2-HG, 2-hydroxyglutarate; PHDs, prolyl hydroxylases; eLF-4E, eukaryotic initiation factor 4E; 4E-BP1, 4E binding protein 1; BCR, B cell receptor.

HIF-1α activation resulted in global translation inhibition coupled with deregulation of mitochondrial function during hypoxic stress in DLBCLs. However, the translational inhibition by HIF-1α is not complete, and the expression levels of hypoxic targets, such as GLUT1 and HK2, are refractory to translational inhibition in DLBCL cells [24] (Figure 2).

### 3.2. MYC

The *MYC* oncogene, which plays a crucial role in several human cancers, including B- and T-cell malignancies, is regarded as a master regulator of cell metabolism and proliferation [116]. As a transcription factor encoded by *MYC*, c-Myc (MYC) can associate altered cellular metabolism with tumorigenesis. Most metabolic genes are housekeeping genes, which have an open chromatin state, and their transcription extension is also enhanced by c-Myc [117].

In lymphoma, MYC activation occurs through various molecular processes, including mutations, amplification, translocations, altered intracellular localization of the MYC protein, and miRNA-dependent mechanisms [118,119,120]. The oncogenic HSP90, which optimizes several MYC metabolic programs, is indispensable to fulfill biomass, energetic, and secretory demands of B-cell lymphomas and, specifically, to support the metabolic process driven by MYC [121]. MYC overexpression, caused by gene rearrangements and mutations, has been observed in various hematological malignancies, especially in invasive B-cell lymphomas, such as DLBCLs and BLs [122,123,124]. MYC protein, the primary regulator of the Warburg effect, regulates HK2 and PDK1 genes cooperatively with HIF-1α. In a BL cell model, the expression level of genes involved in glucose metabolism was decreased after the loss of MYC function [110,125]. In BLs, the MYC protein not only promotes glucose uptake that induces aerobic glycolysis, but it also enhances glutamine uptake and glutamine catabolism by up-regulating mitochondrial GLS [41] (Table 1). In addition, MYC promotes cell proliferation in malignancies through various biosynthetic pathways, particularly ornithine and polyamine biosynthesis pathways [63,64,126]. Through the downregulation of GLS via the upregulation of miR-23b* and an increase in the expression of the enzymes of proline biosynthesis from glutamine, MYC suppresses the expression of the mitochondrial tumor suppressor POX/PRODH [41]. In addition, by upregulating the key enzyme phosphate cytidylyltransferase 1 in DLBCLs, MYC promotes choline metabolism and inhibits mitophagy-dependent necroptosis [127] (Figure 2).

### 3.3. The PI3K/mTOR Pathway

Through activating mutations, gene amplifications, or rearrangements in *PIK3CA* and receptor tyrosine kinases or inactivating mutations in tumor suppressor genes, the PI3K/AKT/mTOR pathway, which regulates cell metabolism, proliferation, apoptosis, and autophagy, is altered in several malignancies [128,129,130,131].

PI3K is a lipid kinase that participates in intracellular signal transduction. Multiple pathways mediated by PI3K-δ and/or PI3K-γ induce cell survival, proliferation, and differentiation in hematologic malignancies [7,132,133]. AKT, a downstream effector of PI3K, is an essential inducer of the glycolytic phenotype, which maintains the survival of cancer cells that depend on glycolysis [134,135]. By up-regulating GLUT1 expression, activating glycolytic enzymes, modulating HK2 expression, and activating and interacting with mitochondria, AKT stimulates glucose uptake and glycolysis [136]. mTOR is a serine/threonine kinase downstream of PI3K-AKT that activates the transcription of multiple genes involved in cancer cell metabolism through mTOR complexes 1 and 2 (mTORC1 and 2) [137]. mTORC1, which is sensitive to rapamycin and environmental alterations [138], is a vital regulator controlling a range of metabolic processes, including glycolysis and mitochondrial metabolism [139,140]. NHLs, characterized by enhanced metabolic activities, are highly associated with aberrant activation of the PI3K/AKT/mTOR signaling pathway [141]. Activation of the PI3K/AKT/mTOR pathway is recurrent in both B-cell lymphomas and TCLs and appears to be related to p53, HIF-1α, and MYC [36,37,38,39,128,142]. Thus, pharmacologic inhibition of the PI3K/AKT/mTOR pathway can be employed for lymphoma therapy (Table 1, Figure 2).

### 3.4. p53

Tumor suppressor p53 regulates numerous signaling pathways in response to diverse cellular stresses, including DNA damage, loss of normal cell contact, abnormal oncogenic events, and hypoxia in some normal cellular processes [143,144].

In NHLs, p53 inhibits glucose uptake by inhibiting the NF-κB pathway and regulating the flux of glucose in glycolysis. Loss of function of p53 gives rise to the activation of GLUT-3 transcription through the NF-κB pathway [145]. In addition, p53 can activate the synthesis of cytochrome c oxidase-2, resulting in the accumulation of ATP and providing negative feedback to the glycolysis pathway. In addition, the p53-inducible enzyme TIGAR can also give negative feedback to glycolysis activity [146].

TP53 is one of the most important factors correlated with dismal survival and mediates cell resistance to chemotherapy in patients with DLBCL. More than 20% of patients with DLBCL have *TP53* mutations, most of which can disrupt protein function and lead to disease progression [147,148]. The adverse outcomes of patients with DLBCLs who exhibit *MYC* rearrangement, MYC expression alone, or concurrent BCL2 expression can be augmented by *TP53* mutations [149] (Figure 2).

### 3.5. AMPK

AMPK, a highly conserved Ser/Thr protein kinase complex composed of catalytic α, regulatory β, and γ subunits, plays a crucial role as a master regulator of cellular energy homeostasis [150]. AMPK deregulates aerobic glycolysis and tumor cell growth in vivo. Blocking AMPK signaling actives the metabolic reprogramming of cancer cells and induces normoxic HIF-1α stabilization, resulting in the Warburg effect and affecting tumor progression in vivo [151]. Defective AMPKα signaling leads to the rewiring of metabolic pathways to favor cell growth pathways, resulting in the functioning of a tumor-suppressor pathway [152,153]. High expression levels of two AMPKβ subunits are significantly associated with prolonged five-year OS in patients with NHLs [154]. In c-Myc transgenic mice, deletion of the AMPKα1 isoform promoted B-cell lymphoma [151]. In addition, the reduction of the AMPKβ1 subunit promotes the development of p53-mutant TCLs [155] (Figure 2). In contrast, AMPK activators (metformin and 5-aminoimidazole-1-β-4-carboxamide-1-β-D-ribofuranoside [AICAR]) were required for capsaicin-induced mTOR complex 1 (mTORC1) inhibition, BCL2 downregulation, and Bax upregulation in osteosarcoma cells [156]. AMPK activation could block lymphoma cell growth via inhibition of the mTOR pathway and the induction of autophagy [157]. For instance, high-dose metformin, which plays an important role in B-cell development, could influence the AMPK signaling and reduce ATP production and inhibit cell proliferation in DLBCL [158]. AICAR had a selective antitumor activity in MCL cell lines. Moreover, AICAR was highly synergistic with rituximab both in vitro and in vivo [159]. Aside from activating AMPK, AICAR can also inhibit mTORC1. When combined with a mTORC1 inhibitor, AICAR enhances the efficacy of rapamycin, which indicated a synergistic effect [160]. In solid tumors, combined treatment of rapamycin and AICAR showed an additive effect on tumor size reduction compared with use of each drug alone [161], which might be theoretically possible to imitate in lymphoma therapy. Therefore, AMPK signaling, regardless alone or combined, might be a potential target in the treatment of DLBCL.

### 3.6. SIRT4

SIRT4, a sirtuin localized in the mitochondria, is a tumor suppressor present in various tumor models. The positive expression of SIRT4 in B-cell lymphoma downregulates glutamine uptake and inhibits cell growth, whereas deletion of SIRT4 in the Eµ-myc models up-regulates glutamine consumption and accelerates tumorigenesis [162]. 

### 3.7. EBV

EBV infects almost every adult with limited symptoms. However, in the state of immune suppression, EBV can cause lymphomas [163]. EBV infection is supposed to participate in the development of a variety of malignant lymphomas and lymphoproliferative disorders (LPDs), such as HL, BL, some types of DLBCL, primary effusion lymphoma, EBV-positive T-cell LPDs, and so on [164]. It has been estimated that nearly half of all HLs contain EBV DNA. Latent membrane protein (LMP) 2A, one of expressed proteins, plays an important role in enabling EBV and establishes a lifelong latent infection and the development of EBV-associated diseases [165]. Epstein–Barr nuclear antigen 2 (EBNA2), together with its key host target MYC, induce metabolism pathways needed for B cell remodeling and proliferation [166]. EBV infection can induces glucose uptake, OXPHOS, purine and pyrimidine, amino acid, and fatty acid biosynthesis pathways in lymphoma development [167]. EBV replication induces B cell expansion in germinal center, actives cytidine deaminase, and MYC translocation, which is essential to the development and progression of BL [168]. Lactate export mediated by MCTs, one part of the Warburg effect, is crucial for continued cell proliferation. EBV infection of B lymphocytes could directly promote the induction of MCT1 and MCT4 through the viral proteins EBNA2 and LMP1 [163]. Dual MCT1/4 inhibition induced lymphoblastoid cell death through the electron transport chain complex I inhibitors [163]. Pharmacological inhibition of lactate export program could render lymphoma cells hypersensitive to metformin and phenformin (electron transport chain complex I inhibitors), which might be a therapeutic approach for targeting EBV related lymphomas [163]. Ectopic expression of LMP1 in EBV-mediated B-cell growth transformation induced FASN [169]. Targeting lipogenesis might be an attractive strategy in treating LMP1-positive EBV-associated lymphomas. NF-κB activation is typical of transformed B lymphocytes, including herpes virus transformed lymphoblasts. Inhibition of the NF-κB pathway in the EBV transformed B-cells lowers glucose uptake and triggers its autophagy-induced death [170]. Besides, EBV infected B cells show upregulation of HIF-1α, MYC, p53, PI3K/mTOR, and AMPK signaling pathways [164], which are all dysregulated in lymphoma metabolic reprogramming.

## 4. Oncometabolites in Lymphoma

Oncometabolites, found in serum, plasma, urine, saliva, and tumor tissue samples, can be evaluated as cancer biomarkers. They refer to metabolites in tumor cells that are significantly upregulated compared with those in normal cells [171]. Oncometabolites are identified based on oncogenic driver mutations of genes encoding some metabolic enzymes [172]. A major mode of action of these oncometabolites is to act as competitive inhibitors of metabolite-dependent enzymes due to their structural similarity to intermediate metabolites [171]. Therefore, mitochondria have a strong effect on controlling the spatial structure of chromatin and give rise to the occurrence of tumors by producing oncometabolites.

### 4.1. Glycolytic Enzymes

Recent studies have shown that increased GAPDH expression can induce NF-κB-dependent HIF-1α activation and can play a crucial role in the angiogenesis and invasiveness of NHLs [22]. In ALCL, the enzymatically inactive isoform of PKM2, regulated by ALK, can induce oncogenesis by phosphorylating nuclear STAT3 and regulating the transcription of genes involved in cell survival and proliferation [40] (Table 1).

### 4.2. SDH

Some TCA enzymes, such as succinate dehydrogenase (SDH), can act as classical tumor suppressors. Dysfunction of SDH can result in the accumulation of metabolic intermediate and exhaustion of aspartate. Aspartate generation depends on the activity of pyruvate carboxylase (PC), which catalyzes the carboxylation of pyruvate to oxaloacetate. SDH-defective cells increase PC expression to synthesize aspartate from glucose to manage TCA cycle truncation and produce aspartate [173,174]. Reduced SDH expression was observed in both doxorubicin- and vincristine-exposed Ramos cells (BL cell line), with a prolonged duration in culture, and this increased the intracellular drug concentration [43] (Table 1).

### 4.3. IDH2, D2HG, and D2HGDH

α-KG, an intermediate metabolite of the TCA cycle, coordinates epigenetic plasticity and leads to malignant behavior and tumor cell survival [175,176]. Under mitochondrial dysfunction caused by the TCA cycle or mutations in the genes encoding electron transport chain enzymes, some glutamine-derived α-KGs undergo reductive carboxylation to support redox homeostasis and biosynthesis [177]. Isocitrate dehydrogenase 1 or 2 (IDH1 or IDH2) frequently undergo gene mutations in AML [178,179,180,181,182]. However, in angioimmunoblastic TCLs, IDH2 mutations play a crucial role in the pathogenesis by increasing DNA hypermethylation of gene promoters [44,45] (Table 1). Recurrent somatic point mutations in the coding regions of IDH1 and IDH2 generate 2-hydroxyglutarate (2HG) through the downregulation of α-KG expression. D-2HG is the exclusive 2HG stereoisomer produced by IDH1 and IDH2 mutants, which can accumulate in high concentrations and inhibit α-KG-dependent dioxygenases, including enzymes involved in DNA and histone demethylation, leading to hypermethylation of CpG islands and histones in DNA [183,184,185,186,187,188,189,190]. D2-HG can be transformed into α-KG by D2-hydroxyglutarate dehydrogenase (D2HGDH). By elevating α-KG levels, wild-type D2HGDH affects histone and DNA methylation and HIF-1α hydroxylation, whereas mutant-type D2HGDH has the opposite effect in DLBCLs [46] (Table 1).

## 5. Therapeutic Strategies Targeting Metabolism

In the past decade, progress in targeting lymphoma metabolism therapeutically has been limited. Only a few metabolism-based drugs for lymphoma have been successfully developed, including those which already have been approved for clinical application and ongoing clinical trials (Table 2).

### 5.1. Targeting Metabolic Pathways

#### 5.1.1. Targeting Glucose Metabolism

Pharmaceutical inhibition of glycolytic enzymes may provide a novel therapeutic strategy for NHLs. HK2 induces the conversion of glucose to glucose-6-phosphate, which is the first step in glycolysis. Two-deoxyglucose (2-DG), an analog of glucose, once bound and phosphorylated by HK2, is not further metabolized, thereby suppressing HK2 [191]. In NHL cell lines, 2-DG can inhibit cell proliferation both under hypoxia and normoxia (Table 3). When combined with methylprednisolone, 2-DG synergistically inhibits cell proliferation by downregulating HIF-1α and c-Myc [192]. Because 2-DG-induced toxicity is regulated by members of the BCL2 family, it can be enhanced by antagonizing BCL2-positive lymphoma cell lines. 2-DG can also induce GADD153/CHOP expression, which is a marker of endoplasmic reticulum stress and an activator of Bim [193]. A glycolysis inhibitor that targets Mcl-1 can restore the sensitivity of lymphoma cells (primary Ramos cells or Eµ-myc) to ABT-737-induced apoptosis [52]. In MCL cells, 2-DG and glucose restriction via anti-glycolytic drugs inhibit TRAIL-induced cell death, indicating that mitochondrial metabolism directly regulates the sensitivity of tumor cells to apoptosis [194]. 3-bromopyruvate (3-BrPA) cannot only restrain tumor glycolysis acting through the hexokinase step, but also hampers mitochondrial ATP production. Both in vitro and in vivo, 3-BrPA demonstrated a significant positive tumor response in Raji-lymphoma-bearing mice [195,196] (Table 3). With augmented induction of apoptosis, 3-bromopyruvate (3-BP), a pyruvate inhibitor and brominated derivative of pyruvate, can inhibit metabolism and survival in Dalton’s lymphoma (DL) cells [197] (Table 3). FX11, a competitive small-molecule inhibitor of LDHA, inhibits cell proliferation and induces death in P493 BL cells by reducing ATP levels and inducing significant oxidative stress [198,199] (Table 3). Interestingly, in lymphoma-bearing mice, α-tocopherol, the most active component of vitamin E, also contributes to keeping cell proliferation in check by downregulating LDHA, PKC-α, and c-Myc expression [199] (Table 3). Inhibition of MCT1 using AZD3965 that blocked lactate efflux led to the accumulation of glycolytic intermediates in vitro and significant downregulation of tumor proliferation in vivo in the Raji BL model. Moreover, when combined with doxorubicin or rituximab, enhanced cell proliferation inhibition and cell death were observed in vitro and in vivo [200,201]. Dichochloroacetate (DCA), a by-product of drinking water disinfection, blocks phosphorylation of PDK at the mitochondrial membrane level, and thus, glycolysis is downregulated due to the activation of the PDH [202,203] (Table 3). Accompanied by modulation of glycolysis and expression of HIF1-α, DCA inhibited cell survival in a DL mouse model [204]. Taken together, these data suggest that glycolytic enzymes with deregulated expression levels are potential biomarkers and have become potential therapeutic targets in NHLs. Tigecycline, approved by FDA, is selectively toxic to OxPhos-DLBCL cell lines and primary tumors and can pharmacologically disturb the mitochondrial translation pathway (Table 3). These findings indicate that the mitochondrial translation pathway is a potential therapeutic target for these tumors as well [56].

The presence of IDH1 or IDH2 mutations and accumulation of 2-HG may result in dependence on specific pathways and the introduction of therapeutic vulnerability. For instance, tumors that harbor IDH mutations are more sensitive to electron-transport-chain inhibitors [205], hypomethylating agents [190], depletion of the canonical coenzyme NAD^+^, and chemoradiotherapy [206]. Pharmacological molecules targeting mutant IDH1 and IDH2 enzymes are being explored and assessed for antitumor efficacy.

**Table 3:** Targeting lymphoma metabolism via metabolic enzymes, metabolite depletion, and/or signaling pathways.

Pathway	Compound	Application	Development Stage	Ref
HK2	2-DG	B-NHL	cell lines	[192]
	3-BrPA	BL	cell lines, mice	[195,196]
Pyruvate	3-BP	T-NHL	mice (DL)	[197]
LDHA	FX11	BL	cell line	[198,199]
	α-Tocopherol	T-NHL	mice (DL)	[199]
PDK	DCA	DL	mice	[202,203]
Mitochondrial protein translation	Tigecycline	OxPhos-DLBCL cell lines	FDA-approved	[56]
Glutaminase	BPTES	BL	mice	[47]
Glutamine uptake	L-asparaginase	NHL	cell lines	[207]
SHMT1/2	SHIN1	DLBCL	cell lines	[33]
FASN	orlistat	MCL	cell lines	[86,208]
		T-NHL	cell lines, mice	[209]
	C75	DLBCL, PEL, and B-NHL	cell lines	[84,85]
NA	BaP	BL	patients	[210]
PPARα	Fenofibrate	B-NHL	mice	[211]
Choline kinase	CK37	T-NHL	mice	[42]
HIF-1α	PX-478	PEL	cell lines	[115]
	PCI-24781(HDACi)	DLBCL	Phase 1/2	[212]
	SAHA(HDACi)	B-NHL	cell lines, mice	[213]
MYC	10058-F4	DLBCL	cell lines	[214,215,216]
PI3K	LY294002	B-NHL	cell lines	[217]
	AZD8835	ABC-DLBCL	cell lines	[218]
AKT	Akti1/2	PEL	cell lines	[219]
	AZD-5363	PTEN-deficient DLBCL	cell lines, mice	[218]
	MK-2206	ABC-DLBCL	mice	[220]
	NaB (HDACi)	BL	cell lines	[221]
mTOR	Rapamycin	ALCL, NHL	cell lines	[36,131]
mTOR C1/2	AZD-2014	MCL	cell lines	[222]
Dual inhibitor of PI3K and mTOR	NVP-BEZ235	PEL	mice	[223]
	PF-04091502	PEL	cell lines	[219]
	Bimiralisib (PQR309)	DLBCL, MCL, SMZL, CLL, HL, and ALCL	cell lines, mice	[224]
AMPK	AICAR	MCL, SMZL, FL, and CLL	cell lines, mice and patients	[159,225,226,227,228,229]
	Metformin	B and T-NHL	cell lines, mice	[157,230]
	Phenformin	PTEN-deficient T-cell lymphomas	cell lines	[230]

Abbreviation: HK2, hexokinase 2; LDHA, lactate dehydrogenase A; PDK, pyruvate dehydrogenase kinase; SHMT1/2, serine hydroxymethyltransferase 1/2; FASN, fatty acid synthase; PPARα, peroxisome proliferator-activated receptor alpha; HIF-1α, hypoxia-inducible factor 1 alpha; PI3K, phosphatidylinositol 3-kinase; mTOR, mammalian target of rapamycin; AMPK; DG, deoxyglucose; BrPA, bromopyruvate; BP, bromopyruvate; DCA, dichloroacetate; BPTES, bis-2-(5-phenylacetamido-1,2,4-diathiazol-2-yl) ethyl sulfide; SHIN1, serine hydroxymethyltransferase 1 inhibitor; HDACi, histone deacetylase inhibitor; SAHA, suberoylanilide hydroxamic acid; AICAR, 5-aminoimidazole-4-carboxamide-1-β-D-ribofuranoside; B-NHL, B-cell non-Hodgkin lymphoma; BL, Burkitt lymphoma; T-NHL, T-cell non-Hodgkin lymphoma; DL, Dalton’s lymphoma; DLBCL, diffuse large B-cell lymphoma; MCL, mantle cell lymphomas; PEL, primary effusion lymphoma; ABC, activated B cell; SMZL, splenic marginal zone lymphoma; FL, follicular lymphoma; CLL, chronic lymphocytic leukemia; ALCL, anaplastic large-cell lymphoma.

#### 5.1.2. Targeting Amino Acid Metabolism

Glutamine metabolism plays a crucial role in cell survival and proliferation under glucose deficiency and hypoxia, making it sensitive to the glutaminase inhibitor Bis-2-[5-(phenylacetamido)-1,3,4-thiadiazol-2-yl]ethyl sulfide (BPTES) [47] (Table 3). L-Asparaginase (L-ASNase), which exhibits some glutaminase activity, can hydrolyze extracellular glutamine to glutamate and ammonia and prevent glutamine from entering the cell [231]. In NHL cell lines, L-ASNase exerts cytotoxicity by depriving the cells of glutamine, resulting in the suppression of cell growth and survival [207] (Table 2 and Table 3).

#### 5.1.3. Targeting Lipid Metabolism

Pharmacological inhibition using the FASN-specific inhibitor C75 triggered caspase-dependent apoptosis in DLBCL cell lines [84] (Table 3). FASN, up-regulated in PEL and other types of non-viral B-NHLs in a PI3K dependent manner, is sensitive to C75 as well [85] (Table 3). FASN is also highly and consistently expressed in MCLs. MCL cell lines, in which FASN is highly and consistently expressed, exhibited significant apoptosis when treated with orlistat, an anti-obesity drug, which is also an inhibitor of FASN and is approved by the FDA [86] (Table 3). Orlistat can also interfere with the ubiquitination of NOXA protein and induce apoptosis, thereby offering new strategies to kill bortezomib-resistant MCL cells [208]. TCLs, when treated with orlistat in vitro, manifest tumor-specific inhibition of cell survival and induction of apoptosis [209]. Orlistat-induced tumor growth retardation decreased tumor cell survival and chemosensitization to cisplatin, and a prolonged life span was observed in tumor-bearing mice [209] (Table 3). BaP, a redeployed drug that combines bezafibrate and medroxyprogesterone acetate, whose complete mechanism was not fully understood, was extended to the clinic and demonstrated efficacy with low toxicity in clinical trials involving patients with BL (ISRCTN34303497) [210] (Table 3). Interestingly, recent findings indicate that obesity is associated with an increased risk of developing malignant lymphomas. In wild-type B-cell lymphoma mice, tumor size was significantly associated with the depletion of white adipose tissues (WAT) and elevated levels of lipid metabolites. Thus, tumor growth can be significantly suppressed with PPARα agonists and the lipid-lowering drug fenofibrate [211] (Table 3).

### 5.2. Targeting Oncogenic Regulators

#### 5.2.1. HIF-1α Inhibitors

**Figure 3:**
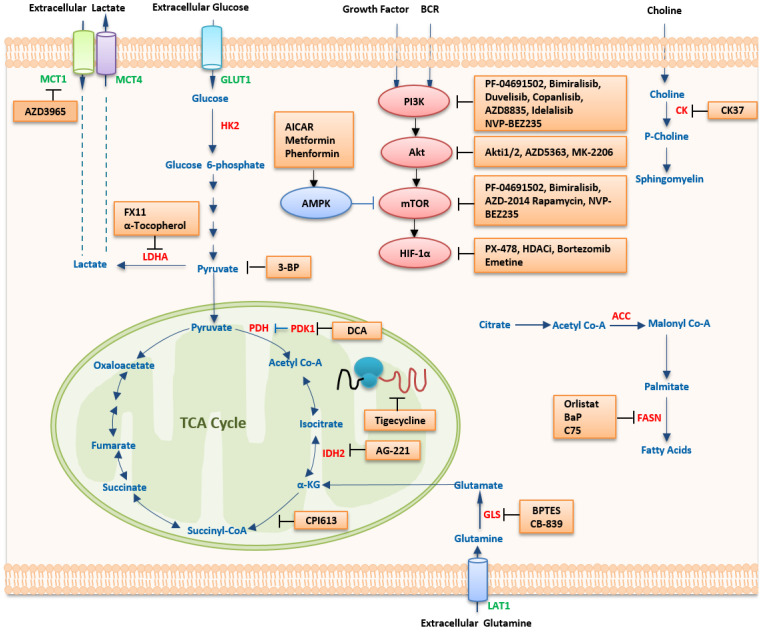
Therapeutic strategies targeting metabolism in lymphoma. Several pathways of the bioenergetic and anabolic metabolism of malignant cells harbor targets for the treatment of cancer. In general, agents that disrupt these pathways would be expected to result in deficiencies in energy and materials needed for cell proliferation and survival, forming the basis for their use as anticancer therapies. Abbreviations: MCT1/4, monocarboxylate transporter 1/4; GULT1, glucose transporter 1; HK2, hexokinase 2; LDHA, lactate dehydrogenase A; PDH, pyruvate dehydrogenase; PDK, pyruvate dehydrogenase kinase; DCA, dichochloroacetate; IDH2, isocitrate dehydrogenase; α-KG, α-ketoglutarate; TCA, tricarboxylic acid; GLS, glutaminase; BPTES, bis-2-(5-phenylacetamido-1,2,4-diathiazol-2-yl) ethyl sulfide; LAT1, L-type amino-acid transporter 1; FASN, fatty acid synthase; ACC, acetyl-CoA carboxylase; HDACi, histone deacetylase inhibitor; HIF-1α, hypoxia-inducible factor 1 alpha; mTOR, mammalian target of rapamycin; AMPK, 5ʹ-AMP-activated protein kinase; AICAR, 5-aminoimidazole-4-carboxamide-1-β-D-ribofuranoside; PI3K, phosphatidylinositol 3-kinase; BCR, B cell receptor; CK, choline kinase. In the metabolic pathways, transporters are in green color, key metabolic enzymes are in red color, metabolites are in blue color, and inhibitors are in black color.

HIF-1α plays an important role in the pathogenesis of lymphoma. Inhibition of HIF-1α may have a therapeutic effect on lymphoma. PX-478 is a small-molecule inhibitor of HIF-1α and inhibits lymphoma cell growth even under normoxia [115] (Table 3). PCI-24781 is a broad-spectrum histone deacetylase inhibitor (HDACi) that can promote the accumulation of HIF-1α and induce initial autophagy of lymphoma cells. Long-term incubation with HDACi can inhibit the expression of HIF-1α [212] (Table 3). Suberoylanilide hydroxamic acid (SAHA, vorinostat), a second-generation HDACi, can inhibit cell proliferation and induce apoptosis by inhibiting the expression of HIF-1α in B-cell lymphoma in vitro and in vivo [213]. Bortezomib is the first proteasome inhibitor used for treating MCLs by suppressing the transcription and expression of HIF-1-specific target genes. Although HIF-1α is not destroyed by bortezomib, heterodimeric HIF-1 cannot transactivate the target genes [232] (Figure 3).

#### 5.2.2. MYC Inhibitors

Most MYC inhibitors are intended to interfere with the MYC–MAX interaction. Because MYC lacks hydrophobic pockets, its structure makes it difficult to target the MYC protein directly. Therefore, inhibition of c-Myc is an attractive strategy to inhibit glucose metabolism and the proliferation of lymphoma cells. In DLBCL cell lines, including GCB and ABC, cell proliferation was inhibited by the c-Myc inhibitor 10058-F4, as a single agent and in combination with 2-DG [217]. However, 10058-F4 is ineffective in vivo due to its poor bioavailability and rapid metabolism and clearance in other cancers [214,215,216] (Table 3). Thus, no small-molecule inhibitor targeting the *MYC* gene is currently tested in clinical trials. Recently, Bhatt et al. cultured DLBCL cells in three-dimensional matrices and discovered a reduced proliferative activity and altered metabolism that is consistent with in vivo tumor cell function [233]. Development of three-dimensional conditions that stabilize tumor cell function towards in vivo phenotype might be helpful for the understanding of lymphoma metabolic process and make progress in relevant research.

A therapeutic approach targeting MYC phosphorylation and degradation is a promising way of treating cancers addicted to high MYC protein levels [234,235,236]. The enzyme 1α/X-box binding protein 1/stearoyl-CoA-desaturase 1 (IRE1α/XBP1/SCD1) axis plays a protective role in counterbalancing anabolism mediated by MYC overexpression. Genetic and pharmacological inhibition of XBP1 leads to MYC-dependent apoptosis, which can be alleviated by exogenous unsaturated fatty acids. In addition, IRE1α inhibition can enhance the cytotoxic effects of standard chemotherapy against BL with MYC overexpression [237].

The oncogenic form of HSP90 optimizes several MYC metabolic pathways, including the production of nucleotides [121]. Given the importance of MYC in driving metabolic reprogramming in lymphoma progress, oncogenic HSP90 inhibitors could reverse the immunosuppressive effects on the lymphoma microenvironment and thus potentially improve efficacy of lymphoma immunotherapy.

#### 5.2.3. The PI3K/mTOR Pathway

##### PI3K Inhibitors

Treatment with the PI3K inhibitor LY294002, either as a single agent or in combination with 2-DG, reduces cell survival, FDG uptake, and cell growth [217] (Table 3). Inhibition of PI3K-α and -δ by copanlisib demonstrated manageable safety and significant efficacy in relapsed or refractory (R/R) indolent lymphoma patients who were heavily pretreated. High response rates were associated with increased expression of PI3K/BCR signaling pathway genes [238] (Table 2). Another PI3K-α/δ inhibitor, AZD8835, demonstrated remarkable potency in ABC-like DLBCL models by inhibiting NF-κB signaling and had a synergistic effect with the Bruton’s tyrosine kinase (BTK) inhibitor ibrutinib both in vitro and in vivo [218] (Table 3). A similar anti-tumor effect was observed in the ABC DLBCL mouse xenograft model treated with a combination of the PI3Kδ inhibitor idelalisib and BTK inhibitor ONO/GS-4059 [220]. Duvelisib, an oral inhibitor of PI3K-δ/γ isoforms, demonstrated acceptable safety and promising clinical activity in patients with R/R TCLs [239]. In vitro, phospho-AKT (pAKT) showed a synergistic effect with duvelisib in TCL cell lines [239] (Table 2 and Table 3, Figure 3).

##### Akt Inhibitors

Akti 1/2, an AKT inhibitor that reduces the rate of lactate production in hypoxic conditions, displays improved cytotoxicity to PEL cells [219]. In combination with the 2-DG, Akti 1/2 demonstrated strong synergistic cytotoxicity toward PEL cells and shifted metabolism from aerobic glycolysis toward oxidative respiration [219] (Table 3). Through the downregulation of MYC, the AKT inhibitor AZD5363 induced apoptosis in PTEN-deficient DLBCLs [218] (Table 3). Combined with the PI3Kδ inhibitor idelalisib, the AKT inhibitor MK-2206 could increase the sensitivity of tumor cells to idelalisib in an ABC DLBCL mouse xenograft [220] (Table 3). In BL cell lines, by regulating AKT phosphorylation and MYC protein expression, cell proliferation was inhibited by HDACi (sodium butyrate, NaB) combined with VP-16, which indicates that the PI3K/AKT pathway is a target of HDACi as well [221] (Table 3). Hypoglycemic agents, such as metformin, phenformin, and the AMPK activator acadesine, have strong antitumor effects on T-cell specific PTEN-deficient (tPTEN−/−) lymphoma cells [230] (Figure 3).

##### mTOR Inhibitors

Rapamycin inhibits the growth of TCLs by decreasing the glycolytic rate and glucose utilization both in vitro and in vivo. Rapamycin-treated cells displayed reduced sensitivity to low-glucose conditions but continued to rely on OXPHOS, which can be reversed by an OXPHOS inhibitor [131] (Table 3). Besides, accumulating evidence indicates that rapamycin may serve as a potential glucocorticoid (GC) sensitizer in lymphomas through genetic prevention of 4E-BP1 phosphorylation [240,241]. Rapamycin also led to a reduction in GLUT1 mRNA expression, FDG uptake, and cell survival of NHL cell lines [36] (Table 3). A dual mTORC1/2 kinase inhibitor AZD-2014 can inhibit cellular energy metabolism via the TCA cycle and further inhibit glycolysis in MCL cells [222] (Table 3, Figure 3).

##### Dual Inhibitors of PI3K and mTOR

Dual targeting of PI3K and mTOR by the inhibitor NVP-BEZ235 led to improved results compared to the application of rapamycin alone in PEL [223] (Table 3). PF-04691502, another dual inhibitor of PI3K and mTOR, which has a synergistic effect with 2-DG, demonstrated increased cytotoxicity in PEL cells under hypoxic conditions or in a glycolytic phenotype [219] (Table 2).

Bimiralisib (PQR309), an oral, novel, selective dual PI3K/mTOR inhibitor, had anti-lymphoma activity as a single agent or in combination with ibrutinib, lenalidomide, and rituximab in vitro. Increased transcripts coding for the BCR pathway can improve the activity of bimiralisib. Bimiralisib showed activity in lymphoma cells with primary or secondary resistance to idelalisib and appeared to be a novel and promising therapeutic compound [224] (Table 3).

#### 5.2.4. AMPK Activators

Activation of AMPK may represent an important therapeutic strategy for lymphoma treatment due to its essential role in its pathway [242,243,244]. Metformin, belonging to the biguanide class of oral hypoglycemic agents, could potentially inhibit cell growth in lymphomas as an AMPK activator both in vitro and in vivo (Table 3). The activation of AMPK promoted by metformin was associated with inhibition of the mTOR pathway without involving AKT [157]. Acadesine (AICAR) is a cell-permeable nucleoside analog that can be metabolically transformed to AICA ribotide (ZMP) by the cells. AICAR can mimic a low-energy state and can regulate the anticancer effects via different mechanisms [242,245,246,247,248]. AICAR has been shown to induce selective cell apoptosis, cell proliferation inhibition, and cell cycle arrest in several hematological malignancies [159,226,227,228,229] (Table 3). AICAR showed an acceptable safety profile in a phase I/II clinical trial involving patients with R/R chronic lymphocytic leukemia [249]. In MCL patients, AICAR showed a cytotoxic effect when combined with rituximab [159]. AICAR triggers the activation of the AMPK pathway, inhibits the downstream mTOR cascade, and forces MCL cells to enter caspase-dependent apoptosis. The selective BH3-mimetic agent ABT-199 targeting BCL2 increases the sensitivity to AICAR in BCL2-overexpressing MCL cells [159,225] (Table 3, Figure 3).

## 6. Potential Metabolic Biomarkers of Lymphoma

Metabolomics has already been used to investigate the pathogenesis of diseases and explore new biomarkers for disease diagnosis, treatments, and prognosis. The PI3K/AKT/mTOR signaling pathway, for instance, has been shown to participate in the cell pro-survival and metabolic reprogramming involving fatty acid metabolism, glycolysis, and tricarboxylic acid cycle in B-cell lymphoma [250]. Wang et al. generated a metabolic gene panel, using 13 metabolism-associated genes (MAGs) in DLBCL-related metabolic pathways, which divided patients with DLBCL into one of two risk groups. The combination of metabolic gene signatures and other prognostic factors showed a superior prognostic power than IPI and other standard prognostic clinical variables [250]. Based on the expression patterns of 92 prognosis associated MAGs mining from public database, He et al. divided DLBCL patients into two metabolic clusters with significantly different prognoses [251]. A prognostic risk model was constructed based on 14 genes selected from 92 MAGs, and then the DLBCL patients were classified into two risk groups, which was superior to the IPI [251]. These nomograms would be useful in clinical practice and future clinical trials. Serum samples from 100 DLBCL patients and 100 matched healthy controls were analyzed by an untargeted mass-spectrometry-based metabolomics platform. The results of this combined study indicate that 2-AG might play a potential role in the pathogenesis and progression in patients with DLBCL [252]. The differences in serum metabolites between BLs and normal mice were analyzed by nuclear magnetic resonance-based metabolomics. Glutamate, glycerol, and choline had a high accuracy of diagnosis, which might provide non-invasive approaches for the diagnosis and prognosis of patients with BL [253]. Serum metabolomics analysis can identify high-risk DLBCL patients with failure of immunochemotherapy, which might provide another novel, non-invasive way for the diagnosis and prognosis of lymphoma [254]. A study on the skin and plasma of CTCL mice indicated that aberrant metabolites and metabolic pathways were essential metabolic features of CTCLs, whereas accumulative cytidine-5′-triphosphate in adjacent non-involved skin tissues led to CTCL further development [255]. Changes in lipid profiles provide new biological insights into how MYC regulates cellular metabolism in MYC-induced lymphoma [256] (Table 4).

## 7. Conclusions and Perspectives

Lymphoma is a hematological malignancy with complex terminology and variable clinical outcomes and continues to be an area of unmet need. Metabolic reprogramming in lymphoma, involving glycolysis, lipid metabolism, and glutaminolysis, is a complicated process regulated by numerous tumor oncogenes, suppressors, and pathways. These regulators mainly target the most crucial enzymes of metabolic pathways, leading to a shift in metabolic phenotypes and accumulation of aberrant metabolites, some of which are related to the biological behavior of lymphoma cells and may serve as potential molecular biomarkers.

However, the molecular heterogeneity of DLBCL brings great challenges to precision therapy. Over the last two decades, molecular and genetic therapeutics have been used in patients, including those with R/R lymphomas. Studies have indicated that immune checkpoints also play crucial roles in metabolic process. Checkpoint signals, on the one hand, can regulate metabolic process [257]. For instance, PD-L1 can activate the PI3K/AKT/mTOR pathway, enhance glucose uptake, and stimulate glycolysis by the tumor cells [258]. On the other hand, metabolism modulates the tumor response to immune checkpoint inhibitor (ICI). For example, obesity patients are recognized to have enhanced PD-1 expression, which is associated with better efficiency to ICIs in solid tumors [259]. Hence, metabolism itself could be a checkpoint that limits immune-mediated tumor destruction [257]. Glutamine metabolism inhibitors have not only improve the immunosuppressive microenvironment, but also effectively reversed treatment resistance to ICI when combined with an ICI [260]. Therefore, combining metabolic inhibitors with checkpoint inhibitors is expected to improve the efficacy of future treatment landscape of lymphoma. Moreover, metabolic reprogramming is an important characteristic of immune cell activation. Accumulation of lactic acid from tumor glycolysis drives macrophages toward the M2 phenotype, which promotes tumor progression [251]. Signal regulatory protein alpha (SIRPα), expressed on macrophages, triggers a “don’t eat me” signal by interacting with the cluster of differentiation 47 (CD47) and further enables immune evasion for lymphoma cells [261]. Antibodies, fusion proteins, small molecule inhibitors, and peptides targeting the CD47-SIRPα axis, together with PD-1(IBI322, HX009), might be promising immunotherapeutic strategies in future lymphoma therapy [262]. A better comprehension of the clinical relevance of metabolic process and microenvironment would help us overcome the barriers to lymphoma treatment and achieve better efficacy and prognosis.

## Figures and Tables

**Table 2:** Primary approved drugs and ongoing clinical trials targeting lymphoma metabolism.

Agent	Target	Mechanism	Type of Diseases
methotrexate	DHFR	folate to THF conversion	prophylaxis and treatment of CNS lymphoma
IM156	mitochondrial complex I inhibitor	mitochondrial oxidative phosphorylation and NADH oxidation	lymphomas (NCT03272256)
IACS-010759	oxidative phosphorylation inhibitor	mitochondrial oxidative phosphorylation and NADH oxidation	R/R AML (NCT02882321)
AZD-3965	MCT1	mediate the bidirectional transport of lactatein and out of cells	DLBCL, BL (NCT01791595)
CPI-613	mitochondria	oxidative metabolism	T-cell NHL (NCT04217317), R/R BL or HGBCL (NCT03793140)
IM156	mitochondria	complex I	advanced lymphoma (NCT03272256)
IACS-010759	mitochondria	complex I	R/R AML (NCT02882321)
ibrutinib	BTK inhibitor	downstream pro-proliferative kinase of BCR signal	MCL, CLL/SLL, WM, MZL, cGVHD (NCT02169180, NCT04771507, NCT02604511, NCT04212013, NCT05348096)
acalabrutinib			MCL, CLL/SLL, MZL (NCT02213926, NCT04505254, NCT04646395)
rapalogs	mTORC1 inhibitor	rapamycin analogues	MCL
temsirolimus	mTORC1 inhibitor	cell cycle arrest in the G1 phase, inhibits tumor angiogenesis by reducing synthesis of VEGF	MCL (NCT01078142, NCT01180049), HL (NCT01902160), R/R NHL (NCT01281917)
idelalisib	PI3Kδ inhibitor	against PI3Kδ isoforms	iNHL (NCT01282424), HL (NCT01393106), FL (NCT03568929), CLL (NCT03582098)
copanlisib	PI3Kδ and PI3Kα inhibitor	against PI3K-α and PI3K-δ isoforms	iNHL, DLBCL, MCL, PTCL (NCT05217914, NCT04433182, NCT04939272, NCT03877055, NCT03052933)
duvelisib	PI3Kδ and PI3Kγinhibitor	against PI3Kδ and PI3Kγ isoforms	CLL/SLL, DLBCL, PTCL (NCT02004522, NCT04890236, NCT04803201)
umbralisib	PI3Kδ and casein kinase-1 epsilon inhibitor	against PI3Kδisoforms and casein kinase-1 epsilon	MZL, FL, CLL, WM (NCT03919175, NCT03364231, NCT02535286)
L-asparaginase	asparagine	inhibit protein biosynthesis in lymphoblasts	ALL (NCT01518517, NCT00506597), NK/T cell lymphoma (NCT00854425)
Telaglenastat (CB-839)	glutaminase inhibitor	glutamine to glutamate conversion	NHL (NCT02071888), including MCL, WM, TCL
AG-270	MAT2A	production of S-adenosylmethionine	advanced lymphoma (NCT03361358, NCT03435250)
Devimistat (CPI-613)	lipoate analog	mitochondrial oxidative metabolism	R/R T-cell NHL (NCT04217317)

Abbreviations: DHFR, dihydrofolate reductase; MCT1, monocarboxylate transporter 1; BTK, Bruton’s tyrosine kinase; mTOR, mammalian target of rapamycin; PI3K, phosphoinositide 3-kinases; MAT2A, methionine adenosyltransferase 2A; THF, tetrahydrofolate; NADH, nicotinamide adenine dinucleotide (NAD) + hydrogen (H); BCR, B-cell receptor; VEGF, vascular endothelial growth factor; CNS, central nervous system; R/R, relapsed or refractory; AML, acute myeloid leukemia; DLBCL, diffuse large B-cell lymphoma; BL, Burkitt lymphoma; NHL, non-Hodgkin lymphoma; HGBCL, high grade B-cell lymphoma; MCL, mantle cell lymphoma; CLL/SLL, chronic lymphocytic leukemia/small lymphocytic lymphoma; WM, Waldenstrom macroglobulinemia; MZL, marginal zone lymphoma; cGVHD, chronic graft versus host disease; HL, Hodgkin lymphoma; iNHL, indolent non-Hodgkin lymphoma; FL, follicular lymphoma; PTCL, peripheral T-cell lymphoma; ALL, acute lymphocytic leukemia; TCL, T-cell lymphoma.

**Table 4:** Potential metabolic biomarkers in NHLs.

Subtype	Control	Metabolites	Type of Samples	Origin	Method	Clinical Relevance	Ref
DLBCL	healthy people	2-AG	serum	patients	LC-MS/MS	pathogenesis or progression	[252]
BL	Normal mice	glutamate, glycerol, and choline	serum	mice	NMR and MS	diagnosis and prognosis	[253]
Refractory or early relapse DLBCL patients	cured patients	↑lysine, arginine, cadaverine, 2-HB	serum	patients	NMR	high-risk of failing to immunochemotherapy	[254]
CTCL	control samples	↑GLT ↓adenosine monophosphate ↑CTP ↑prostaglandins, pyrimidine, mevalonate pathway ↓tryptophan ↑PRPP	skin and plasma	mice	UHPLC-QTOF	progress of carcinogenesis, leads to CTCL further development.	[255]
MYC-induced lymphomas	normal tissue	↑glycerophosphoglycerols, cardiolipins and monounsaturated fatty acids	tissue and cells	patients and cell lines	DESI-MSI	MYC regulates cellular metabolism in cancer	[256]

Abbreviations: DLBCL, diffuse large B-cell lymphoma; BL, Burkitt lymphoma; CTCL, cutaneous T-cell lymphoma; 2-AG, 2-arachidonoylglycerol; 2-HB, 2-hydroxybutyrate; GLT, L-glutamate; CTP, cytidine-5′-triphosphate; PRPP, 5-phospho-α-D-ribose 1-diphosphate; LC-MS/MS, liquid chromatography-tandem mass spectrometry/mass spectrometry; NMR, nuclear magnetic resonance; MS, mass spectrometry; UHPLC-QTOF, ultra-high-performance liquid chromatography–quadrupole time-of-flight; DESI-MSI, desorption electrospray ionization mass spectrometry imaging.

## Data Availability

Not applicable.
